# Electrophoretic Deposition of Co_3_O_4_ Particles/Reduced Graphene Oxide Composites for Efficient Non-Enzymatic H_2_O_2_ Sensing

**DOI:** 10.3390/ma16031261

**Published:** 2023-02-01

**Authors:** Qian Wang, Yuzhe Wang, Guiyong Xiao, Xinde Zhu

**Affiliations:** 1 Key Laboratory of Liquid-Solid Structural Evolution and Processing of Materials of Ministry of Education, School of Materials Science and Engineering, Shandong University, Jinan 250061, China; 2 Shandong Engineering & Technology Research Center for Superhard Material, Jinan 250061, China

**Keywords:** reduced graphene oxide, cobalt oxide particles, electrophoretic deposition, non-enzymatic sensing, hydrogen peroxide

## Abstract

In this work, the facile fabrication of Co_3_O_4_ particles/reduced graphene oxide (Co_3_O_4_/rGO) composites on Indium tin oxide (ITO) slide was achieved by an electrophoretic deposition and annealing process. The deposition time and ratio of the precursors were optimized. Structural characterization and chemical composition investigation indicated successful loading of Co_3_O_4_ particles on graphene sheets. When applied as a non-enzymatic H_2_O_2_ sensor, Co_3_O_4_/rGO showed significant electrocatalytic activity, with a wide linear range (0.1–19.5 mM) and high sensitivity (0.2247 mA mM^−1^ cm^−2^). The good anti-interference ability, reproducibility, and long-term stability of the constructed sensor were also presented. The application of Co_3_O_4_/rGO in real sample analysis was evaluated in human urine sample with satisfactory results, indicating the feasibility of the sensor in physiological and medical applications.

## 1. Introduction

Hydrogen peroxide (H_2_O_2_) is a molecule that plays important roles in industrial, pharmacological, and clinical processes [1,2]. On account of its strong oxidizing ability, H_2_O_2_ is often used for sterilization and food production [3]. As a by-product of biochemical reactions during metabolism, the amount of H_2_O_2_ should be regulated, as over-production of H_2_O_2_ in the body can induce diseases such as cancer, Alzheimer’s disease, and cardiovascular disorders [4,5]. The quantitative determination of H_2_O_2_ is important for industrial and medical purposes. Electrochemical H_2_O_2_ sensors immobilized with enzymes were reported with high specificity and sensitivity [6]. However, enzymatic sensors suffer from issues such as high cost, poor recycling ability, complicated procedures, etc. Therefore, increasing attention has focused on non-enzymatic H_2_O_2_ sensing.

Transition metal nanoparticles (Cu, Co, Au, Ag, etc.) and metal oxides (CuO, Cu_2_O, Co_3_O_4_) are attractive materials for non-enzymatic H_2_O_2_ sensing [7].Among these, Co_3_O_4_ seems to be a promising candidate with low-cost, high abundance, minimal surface fouling, and improved selectivity [8], which are advantageous in H_2_O_2_ detection. However, Co_3_O_4_-based catalysts usually exhibit unsatisfactory performance due to the poor conductivity, few active sites or agglomeration problems [9]. In order to enhance the catalytic ability of Co_3_O_4_, researchers have combined Co_3_O_4_ with carbon-based materials such as graphene and carbon nanotubes to form hybrids [10,11]. Graphene, a 2D carbon nanosheets, displays properties such as a high surface area, good electrical conductivity, and chemical stability. In electrochemical applications, reduced graphene oxide (rGO), one derivative of graphene, has drawn much attention, as its functional groups and defect sites can act as active sites for the bonding of nanomaterials and can facilitate electron transfer. Reports have shown that outstanding electrochemical sensing performance was achieved by incorporating Co_3_O_4_ with rGO [12,13]. Various methods have been explored for the preparation of Co_3_O_4_ and rGO composites such as the solvothermal method [14], hydrothermal method [15], or laser irradiation method [16]. Indeed, hydrothermal/solvothermal methods are widely used synthesis strategies for uniform morphology of the material by adjusting experimental conditions and precursors. For instance, Co_3_O_4_ nanowires on 3D graphene were prepared by a hydrothermal procedure at 120 °C for 16 h [17]. Hollow and mesoporous Co_3_O_4_ spheres were synthesized by the surfactant-assisted solvothermal method, with autoclaving at 200 °C for 4 h in absolute methanol [18]. Although the process is simple without expensive experimental facilities such as the irradiation method, the long reaction time and inhomogeneous thermal distribution in the autoclave could affect the evenness and repeatability of materials [19].

Electrophoretic deposition (EPD) has emerged as a cost-effective and facile strategy to fabricate electrochemical catalysts at room temperature [20]. In an EPD-coating process, particles in the colloidal suspensions migrate to the electrode with opposite charge under applied voltage, yielding uniform coatings on various substrates with a controllable deposition process by simply adjusting the deposition parameters such as the applied voltage and deposition time [21]. In addition, the conductivity and the degree of packing density of deposited layers can be controlled well by EPD, which could enhance the efficiency of the electrocatalyst layers [22]. The EPD process takes a relatively shorter time, such as a few seconds or minutes. Prompt EPD also makes the scaling up to large dimensions possible [23]. In previous work, we showed the use of the EPD technique to fabricate carbon-based nanocomposites for non-enzymatic glucose sensing [24]. Herein, we report the facile fabrication of a Co_3_O_4_ particle/reduced graphene oxide (Co_3_O_4_/rGO)-modified ITO interface by one-step EPD and a subsequent annealing process. The Co_3_O_4_ particles/rGO composites were applied to non-enzymatic H_2_O_2_ detection with a wide linear range and feasibility in real sample analysis.

## 2. Materials and Methods

### 2.1. Chemicals

Cobalt (II) nitrate hexahydrate (Co(NO3)_2_·6H_2_O), α-D-glucose, uric acid (UA), ascorbic acid (AA), dopamine hydrochloride (DA), ethanol, and sodium hydroxide were purchased from Aladdin Reagent Co., Ltd. (Shanghai, China) and used as received. Then, 0.1 M phosphate buffer (PBS, pH = 7.4) was prepared using Na_2_HPO_4_ and NaH_2_PO_4_ by adjusting the pH with H_2_SO_4_ or NaOH. All chemicals were of analytical grade and were used as received without any further purification. Graphene (reduced graphene oxide, purity: >98 wt %) was purchased from Chengdu Organic Chemicals Co. Ltd., Chinese Academy of Sciences (Chengdu, China). Indium tin oxide (ITO) slides (10 Ω/sq) were obtained from Zhuhai Kaivo Optoelectronic Technology Co., Ltd, Zhuhai City, China. A urine sample was collected from a healthy colleague in our group. The water used in all experiments was purified with an ultrafiltration system from Ulupure Co.(Chengdu, China, resistivity = 18.25 MΩ.cm). 

### 2.2. Preparation of the Co_3_O_4_/rGO/ITO Interfaces

The Co_3_O_4_/rGO-modified ITO interfaces were obtained through electrophoretic deposition. Prior to modification, the ITO slides were cleaned thoroughly by successive sonication in acetone, ethanol, and deionized water, followed by blow drying. In the typical EPD process, platinum foil (1 × 1.5cm^2^) and an ITO slide (1 × 1.5cm^2^) function as the anode and cathode, respectively, with a parallel distance of 1 cm in the cell. After sonication for 1 h, a suspension of graphene (0.25 mg ml^−1^) and Co(NO3)_2_·6H_2_O (0.5mg ml^−1^) in ethanol was transferred to the cell, and a DC voltage of 50 V was applied for 1–3 min. Then, the interfaces were washed with deionized water and dried in the air, followed by annealing at 400 °C for 1 h under the protection of an argon atmosphere. The interfaces coated with Co_3_O_4_ particles/rGO with different ratios of Co^2+^ vs. graphene (3:1, 1:1, 1:2, 1:3) were deposited under the same conditions unless mentioned otherwise. The electrode modified with rGO or Co_3_O_4_ alone was obtained using the same method in the absence of Co^2+^ salts or graphene. 

### 2.3. Instrumentation

A Rigaku (Tokyo, Japan) DMAX-2500PC X-ray diffractometer (XRD) with Cu Kα radiation was employed to verify the crystalline phases of the samples. The microstructure structure and morphology of the interfaces were obtained by using a thermal field scanning electron microscope (SEM, SU-70, Hitachi, Tokyo, Japan) equipped with an energy dispersive spectrometer. The surface composition and element properties were characterized by X-ray photoelectron spectroscopy (XPS, AXIS SUPRA, Shimadzu, Kyoto, Japan). Raman spectroscopy measurements were performed by a Raman microscope (Renishaw inVia, UK) with a laser excitation of 532 nm.

Electrochemical measurements were carried out using a CHI 660E electrochemical workstation (Chenhua Instrument, Shanghai, China) using the 3-electrode system, in which the Co_3_O_4_/rGO/ITO interface worked as the working electrode, Ag/AgCl as the reference electrode, and the platinum plate as the counter electrode. Cyclic voltammetry (CV) curves were recorded using aqueous solutions of 0.1 M NaOH/0.1 M phosphate buffer (PBS, pH = 7.4) as the electrolyte. The chronoamperometric performances of the Co_3_O_4_/rGO/ITO electrode were performed in stirred electrolyte solutions under N_2_-saturated PBS, with the successive addition of H_2_O_2_. In real sample analysis, urine was diluted by PBS and spiked with a fixed concentration of H_2_O_2_. The response of the proposed sensor towards H_2_O_2_ in the urine sample was recorded. 

## 3. Results and Discussion

### 3.1. Characterization of the Co_3_O_4_/rGO Composites

**Figure 1:**
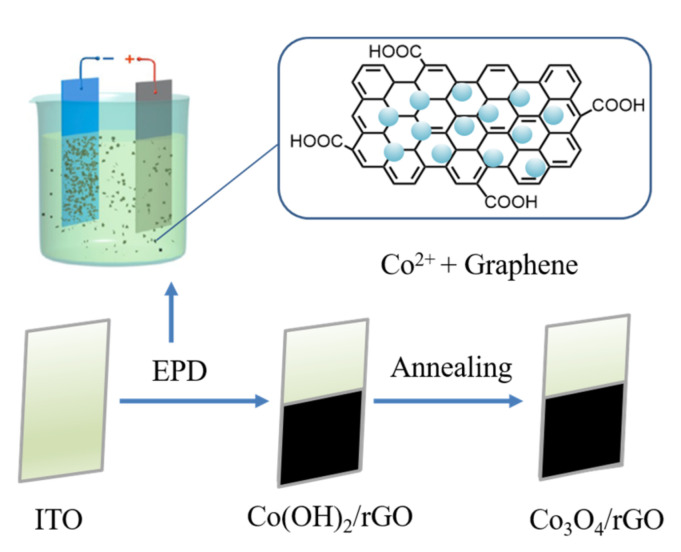
Schematic illustration of the fabrication of the Co_3_O_4_/rGO composites.

The Co_3_O_4_/rGO composites were successfully deposited on the ITO interface via a facile electrophoretic method and subsequent thermal annealing process. As illustrated in Figure 1, positively charged Co^2+^ with graphene moved towards the cathode with the applied voltage of 50 V as the driving force. At the surface of the ITO, the reduction of NO_3_^−^ ions occurred and the generated OH^−^ reacted with absorbed Co^2+^, forming Co(OH)_2_ on the graphene sheets. The coating scheme is as follows [25,26]:

NO_3_^−^ + H_2_O + 2e^−^ → NO_2_^−^ + 2OH^−^ (1)

2H_2_O + 2e^−^ → H_2_ + 2OH^−^ (2)

Co^2+^ + 2OH^−^ → Co(OH)_2_ (3)

**Figure 2:**
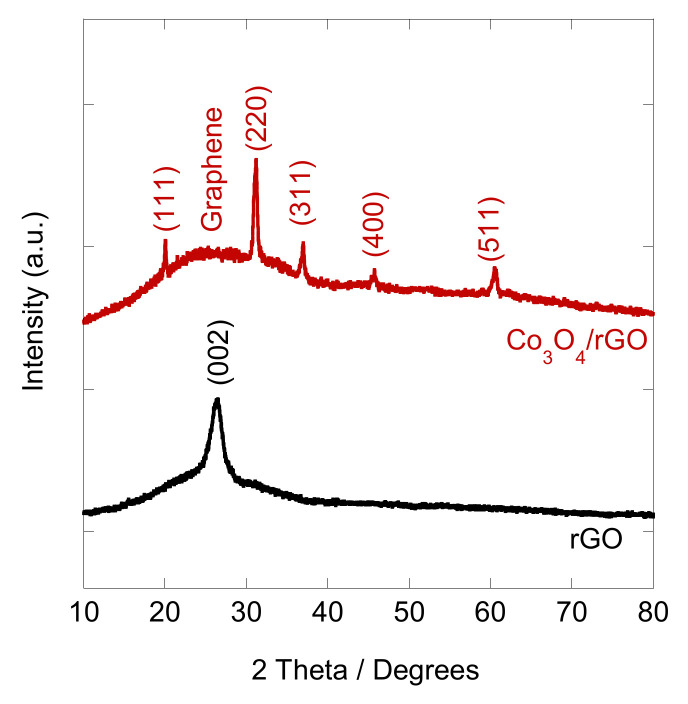
XRD patterns of rGO and Co_3_O_4_/rGO composites.

**Figure 3:**
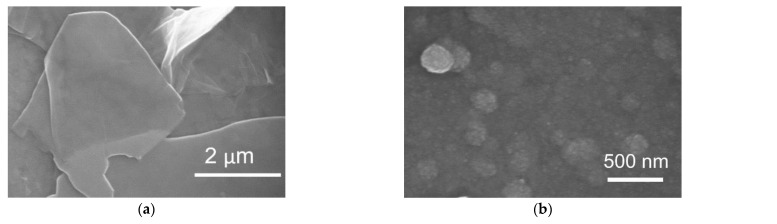

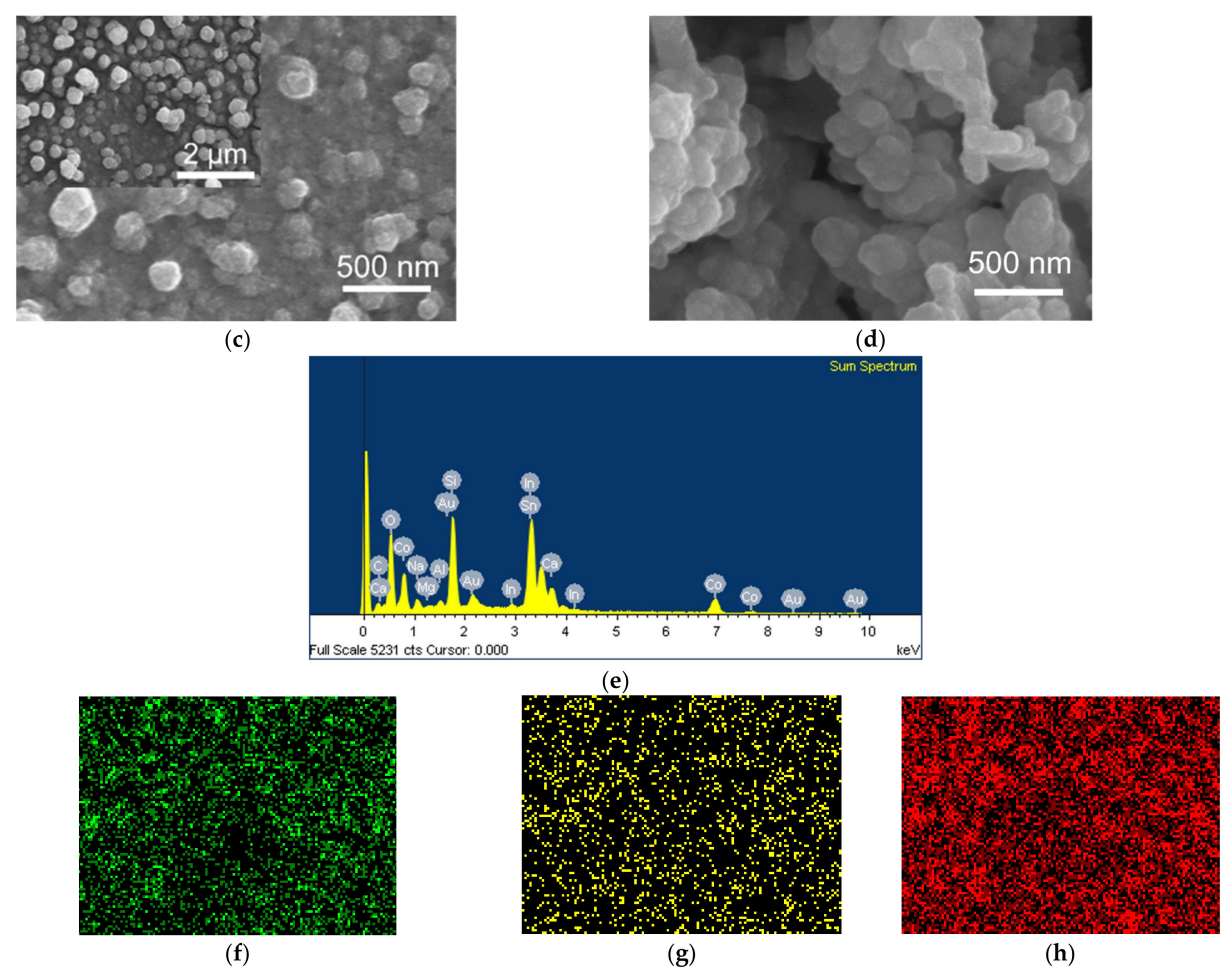
SEM images of (**a**) rGO; Co_3_O_4_/rGO with a deposition time of (**b**) 1 min; (**c**) 2 min (Inset: low magnification image); (**d**) 3 min; EDX results of Co_3_O_4_/rGO (2 min deposition): (**e**)EDX spectrum and EDX elemental mapping (**f**) Co; (**g**) C; (**h**) O.

After the EPD, the coated interfaces were annealed at 400 °C for 1 h in an Ar atmosphere to convert Co(OH)_2_/rGO to Co_3_O_4_/rGO. In order to verify the crystalline structure of the synthesized Co_3_O_4_/rGO composite, XRD analysis was conducted. As shown in Figure 2, the XRD pattern of graphene displayed a peak at 26.4°, corresponding to the (002) plane of graphene. After integration with Co_3_O_4_, diffraction peaks at 20°, 31.2°, 37°, 45.1°, and 60° appeared, which can be ascribed to the respective (111), (220), (311), (400), and (511) planes of Co_3_O_4_ (JCPDS no. 42-1467) [8,25]. The peak of (002) for graphene became broader, indicating that the structure of graphene was restored after EPD with Co [27]. The morphology of the as-prepared Co_3_O_4_/rGO composites was characterized by SEM. EDX spectroscopy analysis was used to examine the chemical composition. In Figure 3a, rGO presents thin layer-by-layer assembly of several nanosheets. After Co(NO_3_)_2_·6H_2_O (0.5 mg ml^−1^) addition, the rGO nanosheets were decorated with Co_3_O_4_ particles in the size of 0.27 ± 0.06 µm (estimated from 100 particles) (Figure 3b–d). The effect of the deposition time on the morphology of the Co_3_O_4_ particles/rGO was evaluated by SEM. Figure 3b–d presents the images of Co_3_O_4_ particles/rGO with varied deposition times of 1 min, 2 min, and 3 min, respectively. It can be seen that after 1 min deposition, several Co_3_O_4_ particles were anchored on the graphene sheets (Figure 3b). The Co_3_O_4_ particles were homogenously distributed with a much higher density when the deposition time was 2 min (Figure 3c). However, the Co_3_O_4_ particles agglomerated as clusters after 3 min deposition, as depicted in Figure 3d. Similar results were found during the EPD process of the Ag nanoparticles [28], the mechanism of which could be explained by Ostwald ripening [29,30]. The EDX spectrum (Figure 3e) performed on Co_3_O_4_/rGO-modified ITO (2 min deposition) comprised signals mainly due to Co, O, C, Si, In, and Sn, consistent with the chemical composition of the material-coated substrate. Signals of Ca, Na, Mg, Al, and Au could be impurities of ITO. The distributions of Co, C, and O were illustrated by EDX mapping in Figure 3f, g, h, respectively, presenting the uniform distribution of Co_3_O_4_ and graphene. The Co atomic concentration was estimated to be 6.04 at%, suggesting the successful deposition of Co. 

**Figure 4:**
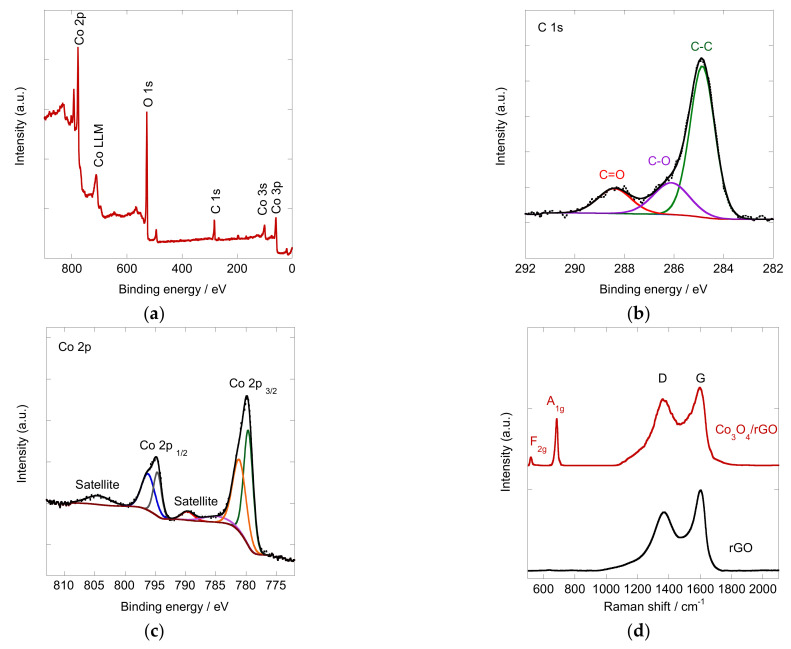
High-resolution XPS of the Co_3_O_4_/rGO composite: (**a**) full spectrum; (**b**) C1s and (**c**) Co 2p spectrum and (**d**) Raman spectra of Co_3_O_4_/rGO (red) and rGO (black).

The chemical composition of the Co_3_O_4_/rGO composite was further investigated by X-ray photoelectron spectroscopy (XPS). As shown in Figure 4a, the full survey of XPS spectra for Co_3_O_4_/rGO clearly demonstrated a series of signals corresponding to the characteristic peaks of Co 3p, Co 3s, C 1s, O 1s, and Co 2p, indicating the existence of Co, O, and C [31]. Figure 4b shows the high resolution of the C 1s spectrum of the Co_3_O_4_/rGO composites. It could be deconvoluted into three peaks at 284.86 eV, 286.11 eV, and 288.40 eV, which are assigned to sp^2^ carbon, C-O, and C=O, respectively, with sp^2^ carbon being dominant [32,33]. The Co 2p core spectrum was deconvoluted and displayed in Figure 4c. The nonsymmetric spin-orbit doublets with shake-up satellites indicate the existence of Co^2+^ and Co^3+^ [34]. The peaks at 779.6 and 794.6 eV with a satellite signal at 789.7 eV were characteristic of Co^3+^, and peaks at 781.1 and 796.2 eV with satellite at 804.4 were attributed to Co^2+^ [35], indicating the formation of Co_3_O_4_.

To gain more insight into the composition of the deposited composites, Raman analysis was conducted. Figure 4d shows the Raman spectra of rGO and Co_3_O_4_/rGO, which displayed the main features of graphene-based materials with D (defects and disorders in the graphitic lattice) and G band (crystalline part of graphene) [36]. The Raman spectrum of rGO exhibited a D band at ~1366 cm^−1^ and a G band at ~1603 cm^−1^. In comparison, the D and G bands of Co_3_O_4_/rGO slightly shifted towards a lower wavenumber and were located at 1361 and 1597 cm^−1^. The red shifts of the D and G bands of Co_3_O_4_/rGO indicated the charge transfer between rGO and Co_3_O_4_ [37]. The I_D_/I_G_ intensity ratio in Raman spectra could provide information on the disorder caused by the defects related to vacancies. The value of I_D_/I_G_ of rGO was 0.73. After integration with Co_3_O_4_, the number increased to 0.86, indicating that the co-deposition of Co_3_O_4_ particles with rGO caused more defects [38], which will serve as active sites for electrocatalytic reactions. Characteristic intensity peaks at 520 and 680 cm^−1^ were observed on the Co_3_O_4_/rGO spectrum, which are attributed to the respective F_2g_ and A_1g_ vibrational modes of Co_3_O_4_ [39,40]. The Raman results showed good accordance with the XPS spectra, indicating the successful incorporation of Co_3_O_4_ on the rGO sheets. 

### 3.2. Electrocatalytic Performance of Co_3_O_4_/rGO in H_2_O_2_ Reduction

**Figure 5:**
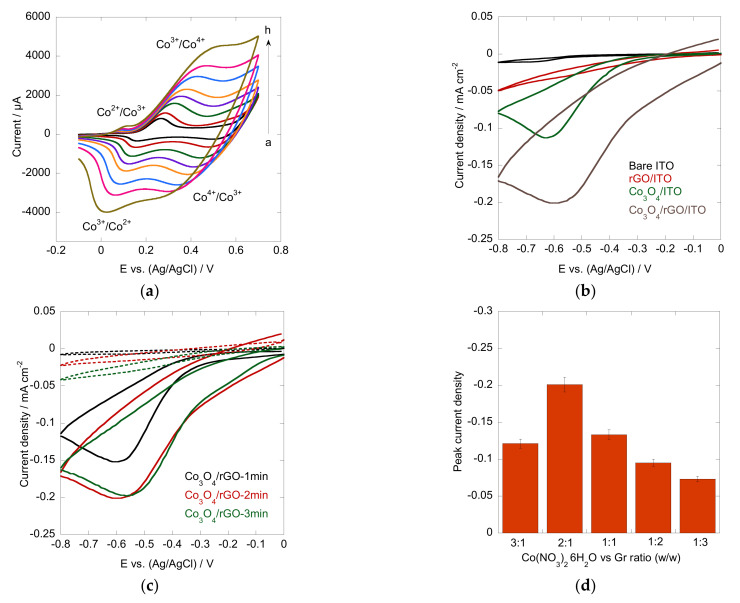
(**a**) CV scans of Co_3_O_4_/rGO in 0.1 M NaOH at different scan rates (a-h: 10–300 mV s^−1^); (**b**) CV of bare ITO (black), rGO/ITO (red), Co_3_O_4_/ITO (green), and Co_3_O_4_/rGO (grey) in the presence of 1 mM H_2_O_2_ in N_2_-saturated 0.1 M PBS buffer (pH = 7.4) at a scan rate of 50 mV s^−1^; (**c**) CV of Co_3_O_4_/rGO-1min (black), Co_3_O_4_/rGO-2min (red), and Co_3_O_4_/rGO-3min (green) in the absence (dashed line) and presence (solid line) of 1 mM H_2_O_2_ in N_2_-saturated 0.1 M PBS buffer (pH = 7.4) at a scan rate of 50 mV s^−1^; (**d**) effect of varied ratios of Co^2+^ vs. graphene.

Prior to the investigation of the electrocatalytic performance of the Co_3_O_4_/rGO interface, cyclic voltammograms (CV) over a potential range of −0.1-0.7 V at various scan rates were recorded in 0.1 M NaOH aqueous solution (Figure 5a). Two reversible redox peaks of Co_3_O/CoOOH, CoOOH/CoO_2_ were observed under alkaline conditions. The reactions can be explained by the following Equations (4) and (5) [17,41]. With the increase in scan rates, the redox peak current increased with positively shifted oxidative peaks and negatively shifted reductive peaks, manifesting a surface-controlled electrochemical process. The typical redox peaks also confirmed the fabrication of Co_3_O_4_/rGO composites.

Co_3_O_4_ + OH^−^ + H_2_O ⇌ 3CoOOH + e^−^ (4)

CoOOH + OH^−^ ⇌ CoO_2_+ H_2_O + e^−^ (5)

The electrocatalytic activity of the Co_3_O_4_/Co composites toward H_2_O_2_ detection was evaluated by CV in N_2_-saturated 0.1 M PBS buffer (pH = 7.4) at a scan rate of 50 mV s^−1^. Figure 5b shows the CV recorded from bare ITO, rGO, Co_3_O_4_, and Co_3_O_4_/rGO composites in the presence of 1 mM H_2_O_2_. Bare ITO and the rGO-modified ITO interface did not show any response to H_2_O_2_ addition under the identified conditions, whereas ITO modified with Co_3_O_4_ displayed a reduction peak at −0.63 V, suggesting that Co_3_O_4_ exhibited notable electrocatalytic activity toward H_2_O_2_ reduction. It is worth noting that a distinct cathodic peak occurred at −0.59 V with higher current density for the Co_3_O_4_/rGO-modified ITO electrode. The highly enhanced electrocatalytic activity and fast electron transfer show the great advantage of Co_3_O_4_/rGO composites for H_2_O_2_ reduction. The improved performance may be attributed to the synergistic effect of the Co_3_O_4_ particles and graphene. The reaction mechanism can be depicted by the following equations [42,43,44]:

Co_3_O_4_/rGO + H_2_O_2_→Co_3_O_4_/rGO-OH_ads_ + OH^−^ (6)

Co_3_O_4_/rGO-OH_ads_ + e^−^→Co_3_O_4_/rGO + OH^−^ (7)

2OH^−^ + 2H^+^→2H_2_O (8)

### 3.3. Optimization of Deposition Conditions

In electrophoretic deposition, the deposition time is an important parameter. Therefore, the effect of the Co_3_O_4_/rGO composites with different deposition times toward H_2_O_2_ detection was evaluated by CV in the absence and presence of 1 mM H_2_O_2_ under otherwise identical conditions (Figure 5c). A higher reduction peak current density was observed at the interfaces after deposition for 2 min (Co:graphene ratio = 2:1 (*w*/*w*)) compared to the interface produced with a deposition time of 1 min, indicating its superior electrocatalytic effect. Although the material deposited in 3 min showed a comparable cathodic peak with a film as that observed at 2 min, the background current decreased. These results well matched the SEM observations, in which 2 min deposition exhibited an appropriate density of Co_3_O_4_ particles without agglomeration. Furthermore, since Co_3_O_4_ particles play an important role in the catalytic reactions, the effect of different ratios of Co(NO3)_2_·6H_2_O to graphene (3:1, 2:1, 1:1, 1:2, 1:3, *w*/*w*) with a deposition time of 2 min was evaluated (Figure 5d). A higher peak current was observed from the interface modified with a Co(NO3)_2_·6H_2_O to graphene ratio of 2:1. Therefore, further electrochemical sensing was carried out on the Co_3_O_4_/rGO-modified electrode with a Co^2+^ to graphene ratio of 2:1 with 2 min deposition.

### 3.4. Amperometric Detection of H_2_O_2_

**Figure 6:**
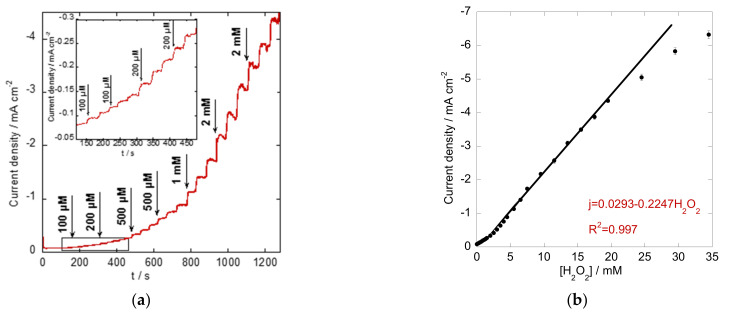
(**a**) Amperometric response of the Co_3_O_4_/rGO-modified electrode polarized at −0.60 V in N_2_-saturated 0.1 M PBS solution with the subsequent addition of H_2_O_2_ (Inset: responses to lower additions of H_2_O_2_); (**b**) the corresponding calibration curve.

The amperometric current–time response of the Co_3_O_4_/rGO-modified electrode was further investigated with continuous injections of different concentrations of H_2_O_2_ into stirred PBS buffer at an applied potential of −0.60 V. As presented in Figure 6a, a staircase response was achieved with increasing concentration of added H_2_O_2_. The current density increased sharply and reached a stable value with a quick response within 6 s. Responses to lower additions are zoomed in as the inset in Figure 6a. In addition, the corresponding calibration curve was acquired by plotting the obtained peak current density against the H_2_O_2_ concentration in Figure 6b. A good linear relationship was obtained from 0.1 to 19.5 mM, with a regression equation as follows: j (mA cm^−2^) =0.0293–0.2247[H_2_O_2_] (mM), R^2^ = 0.997. An estimated sensitivity of 0.2247 mA mM^−1^ cm^−2^ and a detection limit of 10 µM (signal-to-noise ratio (S/N) = 3) were achieved using the Co_3_O_4_/rGO composite-modified electrode. The sensing performance of the proposed electrode showed a comparable wide linear range and high sensitivity to the previously reported H_2_O_2_ sensors based on Co or the carbon-related nanostructures listed in Table 1.

### 3.5. Selectivity, Reproducibility, and Long-Term Stability

**Figure 7:**
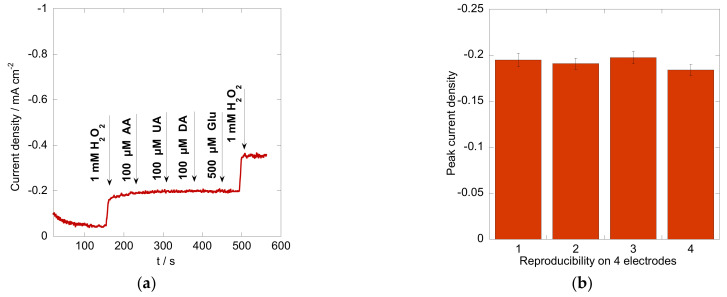
(**a**) The amperometric response of the Co_3_O_4_/rGO-modified electrode exposed to H_2_O_2_ (1 mM), ascorbic acid (AA, 100 µM), uric acid (UA, 100 µM), dopamine (DA, 100 µM), glucose (500 µM), and H_2_O_2_ (1 mM) in N_2_-saturated 0.1 M PBS buffer with an applied potential of −0.60 V; (**b**) Reproducibility analysis based on four electrodes modified with Co_3_O_4_/rGO composites.

The selectivity of the constructed sensor was evaluated by chronoamperometry in the presence of interfering biomolecules. Figure 7a displays the amperometric response of the electrode upon successive additions of 1 mM H_2_O_2_, 100 µM ascorbic acid (AA), 100 µM uric acid (UA), 100 µM dopamine (AA), and 500 µM glucose (Glu) in N_2_-saturated PBS with a biased potential of −0.60 V. No obvious current increase was observed with the injection of the interferences mentioned above, suggesting the outstanding anti-interference ability of the sensor, which is quite important in real sample analysis. Moreover, the reproducibility was examined for four individual Co_3_O_4_/rGO-modified electrodes in 0.1 M PBS (pH = 7.4) in the presence of 1 mM H_2_O_2_ (Figure 7b). A relative standard deviation (RSD) of 2.69% was found, indicating high reproducibility of the modified electrodes. The long-term stability of the Co_3_O_4_/rGO-modified electrode was evaluated by measuring its response to 1 mM H_2_O_2_ before and after storage of 15 days. The current retained 95.3% of its initial response after 15 days, suggesting good storage stability of the sensor.

### 3.6. Real Sample Analysis

In order to test the practical applicability of the Co_3_O_4_/rGO-modified electrode, the sensor was used for the determination of H_2_O_2_ in urine based on the standard addition method. First, 1 mL urine samples were diluted to 10 mL by PBS and spiked with known H_2_O_2_ concentrations. The recoveries of the H_2_O_2_ in Table 2 were determined by recording the current density at −0.6 V. The recovery value of the spiked H_2_O_2_ was in the range of 98–101.5%, indicating feasibility in real sample analysis of the proposed Co_3_O_4_/rGO sensor. 

## 4. Conclusions

In summary, we have demonstrated the easy fabrication of Co_3_O_4_ particles/rGO through electrophoretic deposition and subsequent annealing. The electrocatalytic activity of Co_3_O_4_/rGO-modified electrodes toward enzyme-free H_2_O_2_ sensing was investigated with optimized deposition conditions. The sensor showed a wide linear range from 0.1 to 19.5 mM and a high sensitivity of 0.2247 mA mM^−1^ cm^−2^. Moreover, good selectivity, reproducibility, and time stability were achieved for H_2_O_2_ detection. The possibility of practical applications was also successfully applied by measuring the H_2_O_2_ concentration in human urine samples. Significantly, the synergetic effect of the graphene and cobalt oxide structures facilitated the hybrids, with potential in other electrochemical applications.

## Figures and Tables

**Table 1:** Comparison of the analytical performance of Co_3_O_4_/rGO-modified electrodes with previously reported non-enzymatic H_2_O_2_ sensors.

Electrodes	Linear Range (mM)	Sensitivity (mA mM^−1^ cm^−2^)	Limit of Detection (LOD)	Ref.
Co_3_O_4_/ATNTs	1.27–26.8	0.03953	6.71	[45]
CoS/RGO	0.0001–2.5424	0.002519	0.042	[42]
CoP nanowires	0.01–12	0.0268	0.48	[46]
Co_3_O_4_/NC	0.01–1.4	0.23	1.4	[32]
CoOOH nanosheets	0.004–0.016	0.099	40	[47]
AuPd@GR	0.005–11.5	0.18686	1	[48]
Nf/porous Co_3_O_4_ nanoparticles	0.001–0.3	0.03897	0.24	[49]
Co-Pt/CNTs	0.0002–1.25	0.744	0.1	[50]
rGO/Au NPs	0.02–25	0.04646	20	[51]
Co_3_O_4_/rGO	0.1–19.5	0.2247	10	This work

Abbreviations: ATNTs: anatase titanium dioxide nanotubes; RGO/rGO: reduced graphene oxide; NC: nitrogen-doped carbon; 0.01–1.4; GR: graphene; Nf: nafion; CNTs: carbon nanotubes; Au NPs: Au nanoparticles.

**Table 2:** Determination of H_2_O_2_ in urine samples.

Spiked (mM)	Found (mM)	Recovery (%)	RSD (%, *n* = 3)
1	0.98	98	2.58
2	2.03	101.5	2.05

## Data Availability

All the data are presented in the manuscript.
